# N6-methyladenosine Regulator-Mediated Immune Genes Identify Breast Cancer Immune Subtypes and Predict Immunotherapy Efficacy

**DOI:** 10.3389/fgene.2021.790888

**Published:** 2021-12-17

**Authors:** Meng-Meng Zhang, Yi-Lin Lin, Wen-Feng Zeng, Yang Li, Yang Yang, Miao Liu, Ying-Jiang Ye, Ke-Wei Jiang, Shu Wang, Shan Wang

**Affiliations:** ^1^ Department of Breast Surgery, Peking University People’s Hospital, Beijing, China; ^2^ Laboratory of Surgical Oncology, Beijing Key Laboratory of Colorectal Cancer Diagnosis and Treatment Research, Peking University People’s Hospital, Beijing, China; ^3^ Breast Tumor Center, Sun Yat-Sen Memorial Hospital, Sun Yat-Sen University, Guangzhou, China

**Keywords:** N6-methyladenosine, breast cancer, tumor microenvironment, immunotherapy, prediction

## Abstract

Breast cancer (BRCA) is a heterogeneous malignancy closely related to the tumor microenvironment (TME) cell infiltration. *N*6-methyladenosine (m6A) modification of mRNA plays a crucial regulator in regulating the immune microenvironment of BRCA. Immunotherapy represents a paradigm shift in BRCA treatment; however, lack of an appropriate approach for treatment evaluation is a significant issue in this field. In this study, we attempted to establish a prognostic signature of BRCA based on m6A-related immune genes and to investigate the potential association between prognosis and immunotherapy. We comprehensively evaluated the m6A modification patterns of BRCA tissues and non-tumor tissues from The Cancer Genome Atlas and the modification patterns with TME cell-infiltrating characteristics. Overall, 1,977 TME-related genes were identified in the literature. Based on LASSO and Cox regression analyses, the m6A-related immune score (m6A-IS) was established to characterize the TME of BRCA and predict prognosis and efficacy associated with immunotherapy. We developed an m6A-IS to effectively predict immune infiltration and the prognosis of patients with BRCA. The prognostic score model represented robust predictive performance in both the training and validation cohorts. The low-m6A-IS group was characterized by enhanced antigen presentation and improved immune checkpoint expression, further indicating sensitivity to immunotherapy. Compared with the patients in the high-score group, the overall survival rate after treatment in the low-score group was significantly higher in the testing and validation cohorts. We constructed an m6A-IS system to examine the ability of the m6A signature to predict the infiltration of immune cells of the TME in BRCA, and the m6A-IS system acted as an independent prognostic biomarker that predicts the response of patients with BRCA in immunotherapy.

## Introduction

Breast cancer is the biggest threat to female health worldwide (Siegel et al., 2020). Although comprehensive efforts have been made in breast cancer treatment, including chemotherapy, radiotherapy, and molecular therapeutics, breast cancer continues to be associated with significant mortality in women and an equally substantial socioeconomic burden (DeSantis et al., 2015). Immunotherapy is revolutionizing the therapeutic approach for solid malignancies, and accumulating data indicate that immune checkpoint antagonists such as programmed cell death-1 (PD-1)/programmed death ligand-1 (PD-L1) inhibitors can induce efficacious and durable clinical responses in a proportion of patients with breast cancer, especially metastatic breast cancer (Emens, 2018; Franzoi et al., 2021). A community of epithelial-derived tumor cells mixed with a community of stromal components, referred to as “tumor microenvironment” (TME), is increasingly recognized as indispensable for mammary tumorigenesis. Immune cells, including regulatory T cells (Tregs), myeloid-derived suppressor cells, and B cells, extracellular matrix components, cancer-associated fibroblasts, blood vessels, and cancer-associated adipocytes are critical components of the TME (Deepak et al., 2020). Because of their complexity and heterogeneity, cancer cells can escape immune surveillance by the TME and induce antitumor immune system suppression, drug resistance, and recurrence of breast cancer (Hanahan and Weinberg, 2011). Therefore, focusing on recognizing the composition and the alterations in the molecular signatures of cells in the TME comprehensively may help in identifying the different immune phenotypes of breast cancer and predicting immunotherapeutic responsiveness.

Posttranscriptional regulation of the transcriptome is an important biological process, and over 170 chemical modifications in RNA have been identified to date (Frye et al., 2018). *N*6-methyladenosine (m6A) modification is a dynamic process of RNA posttranscriptional modification and exerts multiple functions in many biology processes, such as transportation, RNA processing, splicing, stability, and degradation of the target RNA (Alarcon et al., 2015; Patil et al., 2016). The m6A machinery is a dynamic and reversible process, including methylation by the methyltransferase complex (“writer”), removal by demethylases (“eraser”), and recognition by m6A-binding proteins (“reader”) (Yang et al., 2018). m6A modification regulates oncogenesis and tumor development, which has been testified (Li et al., 2019; Vu et al., 2019). Overexpression of the m6A reader *YTHDF3* or the m6A demethylase *ALKBH5* may enhance the transcription of m6A-enriched genes in breast cancer, facilitating breast cancer brain metastasis (Zhang et al., 2016; Chang et al., 2020).

Since numerous studies have concentrated on tumor intrinsic cells, the potential role of m6A modification in TME and antitumor immune response has been rarely reported. However, little is known about whether messenger RNA (mRNA) m6A methylation in immune cells is responsible for regulating the TME, which leads to inhibition of immune function and tumor migration. Recent studies have provided some clues. YTHDF1, a well-known m6A-binding protein, undermines the cross-presentation of engulfed neoantigen-specific immunity by interacting with transcripts encoding lysosomal proteases in dendritic cells, suggesting that altered m6A modification may facilitate immune evasion in tumors (Han et al., 2019).

**FIGURE 1:**
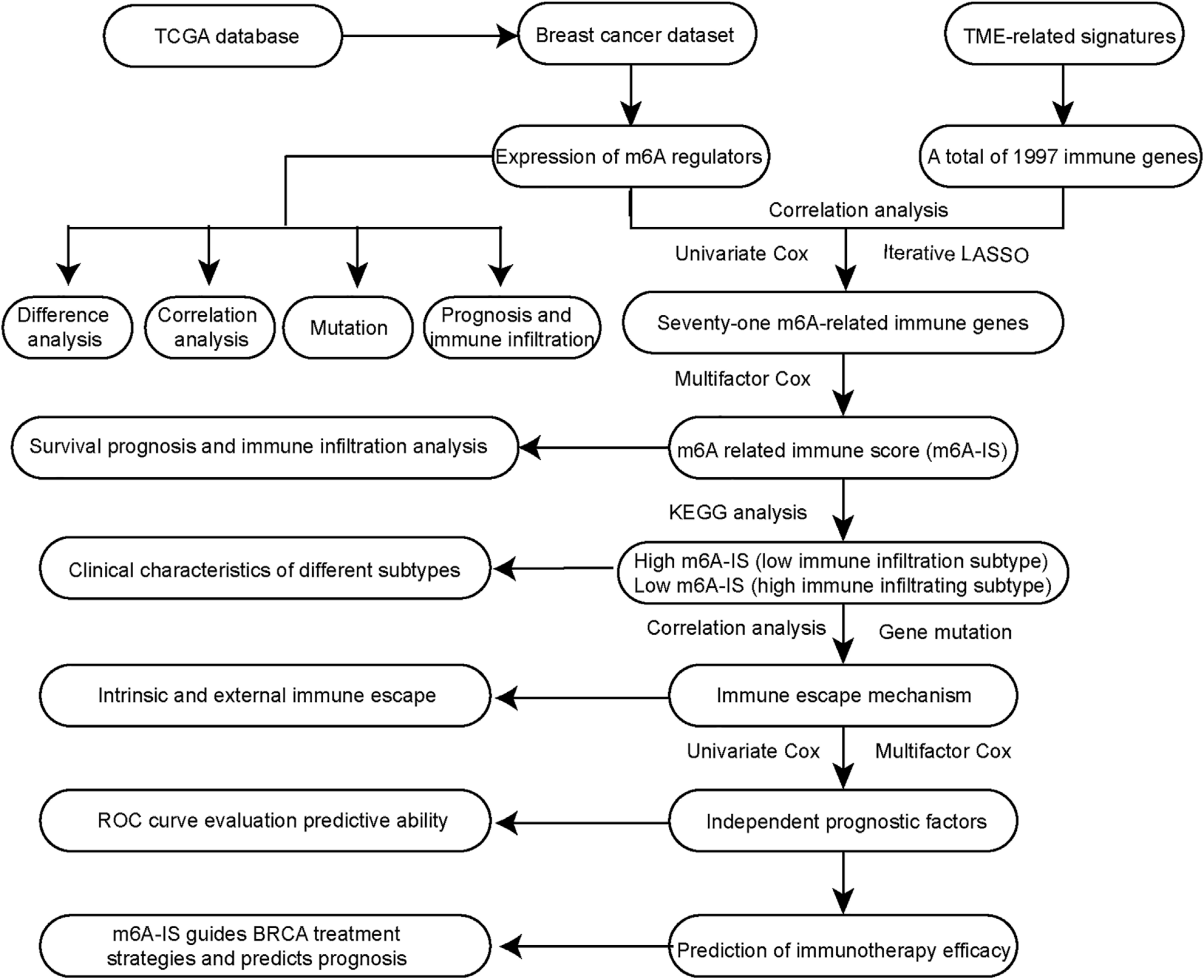
Flowchart of the research.

In our study, we initially identified the characteristics of immune cell types and 24 m6A regulators in the literature. Then, we identified the m6A-related immune genes with TME cell-infiltrating characteristics using Pearson’s correlation analysis. A new m6A-related immune score (m6A-IS) prediction system was constructed based on m6A-related genes to assess the prognosis of breast cancer. Moreover, we showed the relationship between m6A-IS and the response to immunotherapy. Ultimately, a predictive nomogram for survival prediction of individual patients with breast cancer verified that m6A modification is non-negligible in drawing different TME characterizations. A flowchart of our research is shown in Figure 1.

## Materials and Methods

### Data Acquisition

The mRNA expression profile data and DNA mutation data (VarScan2) of breast cancer samples were obtained from The Cancer Genome Atlas (TCGA) database; 1,068 samples with complete prognostic information were included. A total of 1,094 breast cancer samples with complete prognostic information were obtained in the METABRIC cohort from cBioportal (www.cbioportal.org). Transcripts per million data were used for subsequent analysis. The data for a cohort of patients with metastatic urothelial cancer treated with anti-PD-L1 therapy were obtained from the R software package IMvigor210CoreBiologies (IMvigor210, version 1.0.0) (Mariathasan et al., 2018).

### Immune Score and Gene Set Enrichment Analysis

The gene set file “c2.cp.kegg.v6.2” was downloaded from the Molecular Signatures database (MSigDB; https://www.gsea-msigdb.org/gsea/index.jsp). The CIBERSORT (Newman et al., 2015) algorithm was used to evaluate the infiltration levels of immune cells in the sample, and 23 immune cell signatures were also used to evaluate the infiltration state of the TME (Charoentong et al., 2017; Jia et al., 2018). ESTIMATE was used to evaluate the immune and stromal scores of each sample (Yoshihara et al., 2013). Single-sample gene set enrichment analysis (ssGSEA) was used to calculate the enrichment scores of the samples using the GSVA package (Hänzelmann et al., 2013).

### Identification of *N* ^6^-Methyladenosine-Related Immune Genes

We obtained a total of 24 m6A regulators from the study by Zhang et al. (2020). A total of 1,997 immune genes were collected from 184 TME-related signatures (Wang et al., 2021). We identified m6A-related immune genes based on Pearson’s correlation analysis between the expression levels of m6A regulators and immune genes in breast cancer. |Pearson’s correlation coefficient| ≥ 0.5 and *p* < 0.001 were set as cutoff values. A Venn diagram (http://bioinformatics.psb.ugent.be/webtools/Venn/) was used to identify the m6A-related immune genes associated with prognosis.

### Construction of *N* ^6^-Methyladenosine-Related Immune Score

Univariate Cox regression analysis was used to screen the m6A-related immune genes (*p* < 0.05 was set as the cutoff value). Iterative LASSO (least absolute shrinkage and selection operator) was used to screen the m6A-related immune genes for subsequent analysis (Wang et al., 2021). The number of iterations was 500, and genes with a frequency greater than 50 were consensus genes for the iteration LASSO. The order of frequency represents the degree of influence of these features, and these features were then incorporated into the multivariate Cox regression model; the inclusion was stopped when the area under the curve (AUC) value of the receiver operating characteristic curve (ROC) reached its peak. The m6A-related immune genes obtained were used to construct the m6A-IS. The m6A-IS was expressed as follows: m6A-IS = (coefficient mRNA_1_ × mRNA_1_ expression) + (coefficient mRNA_2_ ×expression of mRNA_2_) + …+ (coefficient mRNA_*n*_ × expression mRNA_*n*_). Visualization of the prognostic value of m6A-IS was obtained using a nomogram.

### Survival Analysis

We arranged the m6A-IS from low to high, starting from one low-expressing patient by setting the loop, calculating the corresponding individual *p*-value and hazard ratio (HR), and saving the calculation results. The minimum *p*-value obtained was used to determine the grouping information, and the samples were then divided into two groups. The Kaplan–Meier method estimated the overall survival (OS) curve, and the difference between survival distributions was evaluated using the two-sided log-rank test implemented in the R package survival. The Kaplan–Meier survival curve constructed *via* the R package survmin.

### Statistical Analysis

The limma package in R was used to determine differentially expressed genes in the breast cancer cohorts. The ggplot2 package and ComplexHeatmap package were used to draw heatmaps and other maps. The R package forest plot was used to plot the forest plots. Wilcoxon’s rank-sum test was used to analyze the differences between the two groups. The Kruskal–Wallis test was used to compare differences between three or more groups. The maftools package was used to map the gene mutations. The AUC was quantified using the pROC R package. All statistical *p*-values were two-sided, and statistical significance was set at *p* < 0.05. All analyses were performed using the R software (version 4.0.2).

## Results

### Landscape of *N* ^6^-Methyladenosine Regulators in Breast Cancer

**FIGURE 2:**
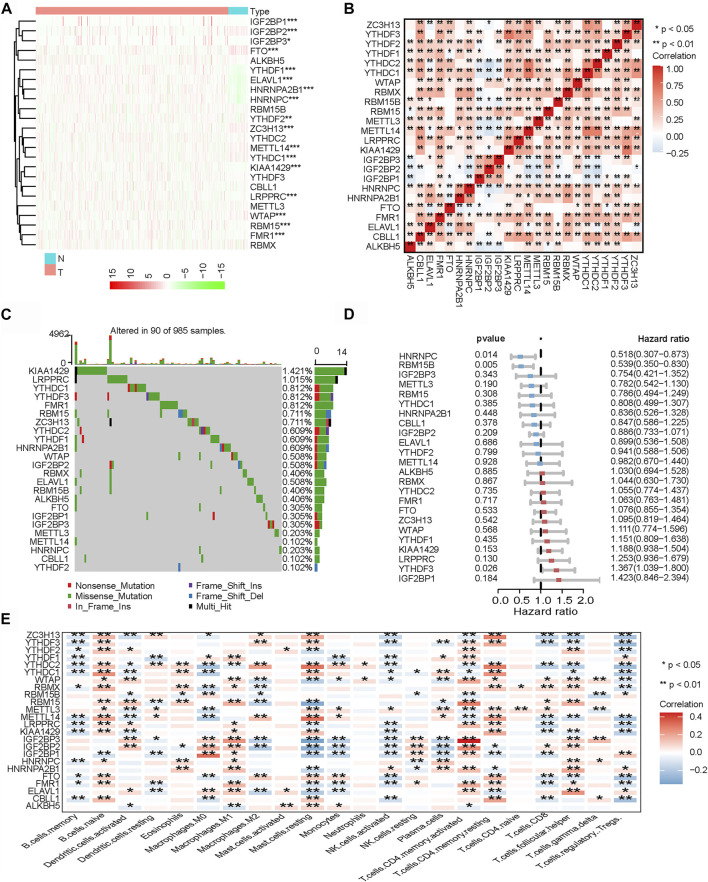
Landscape of *N* ^6^-methyladenosine (m6A) regulators in breast cancer. **(A)** Heatmap of all m6A regulators in breast cancer tissues and normal tissues from The Genome Cancer Atlas (TCGA) database. Each *column* represents individual patients (*blue* indicates normal tissue, *pink* indicates breast cancer tissue; the *darker the red*, the more obvious the upregulation of gene expression, and the *darker the green*, the more obvious the downregulation of gene expression). **(B)** Co-expression of m6A regulators (*p < 0.05; **p < 0.01; ***p < 0.001). **(C)** Mutation frequency of 24 m6A regulators in 985 breast cancer samples from TCGA-BRCA cohort. **(D)** Univariate Cox regression was performed to analyze the hazard ratio of each m6A-related gene in predicting overall survival in breast cancer. **(E)** CIBERSORT was used to analyze the component correlation between the 22 immune cells [tumor microenvironment (TME) infiltrating cells] and 24 m6A regulators (*p < 0.05; **p < 0.01; ***p < 0.001).

In the aggregate, 24 m6A regulators were identified in our study. In order to enrich the dysregulated genes and their correlations, the RNA transcriptomic datasets containing the next-generation sequencing (RNA sequencing, RNA-seq) data of 1,109 breast cancer tissues and 113 non-tumor tissues from TCGA project (TCGA-BRCA) was downloaded. Then, we explored the differential expressions in breast cancer tissues and normal mammary tissues in TCGA. Compared with normal tissues, there were 17 m6A regulators that were significantly differentially expressed in breast cancer tissues (Figure 2A). A correlation analysis was performed for the 24 m6A regulators (Figure 2B). The results showed that the expressions of the 24 m6A regulators were significantly correlated. Subsequently, we summarized the frequency of somatic mutations in the 24 m6A regulators in breast cancer. Of 985 samples, only 90 had mutations in the m6A regulators; indeed, there was a very low frequency of mutations (9.14%). Interestingly, we observed few mutations in breast cancer populations (Figure 2C). By performing univariate Cox regression analysis, we identified the affected prognosis of the 24 m6A-associated genes in patients with breast cancer (Figure 2D). Among the 24 m6A regulators, *HNRNPC* and *RBM15B* were identified as significant protective factors for survival (*p* < 0 0.05), and *YTHDF3* was a risk factor for survival (*p* < 0 0.05).

Related research works have shown that the expressions of m6A regulators are associated with the heterogeneity of the TME (Han et al., 2019; Zhang et al., 2020). Therefore, we further analyzed the relationship between the m6A regulators and cell infiltration in the TME. We analyzed the association between 22 types of immune cells (CIBERSORT algorithm) and m6A regulators (Figure 2E). The heatmap showed that most of the significant enrichments were found in immune cells. The infiltration levels of Tregs, activated natural killer (NK) cells, and memory B cells were significantly related to the expressions of most m6A regulators. The infiltration levels of naive B cells, M1 macrophages, and memory CD4 T cells were significantly positively correlated with most m6A regulators. The results showed that the expressions of m6A regulators were significantly correlated with the levels of immune cell infiltration (*p* < 0.05); indeed, they play a non-negligible role in the regulation of the TME in breast cancer.

### Construction of the *N* ^6^-Methyladenosine-Related Immune Score and Analysis of Its Characteristics

**FIGURE 3:**
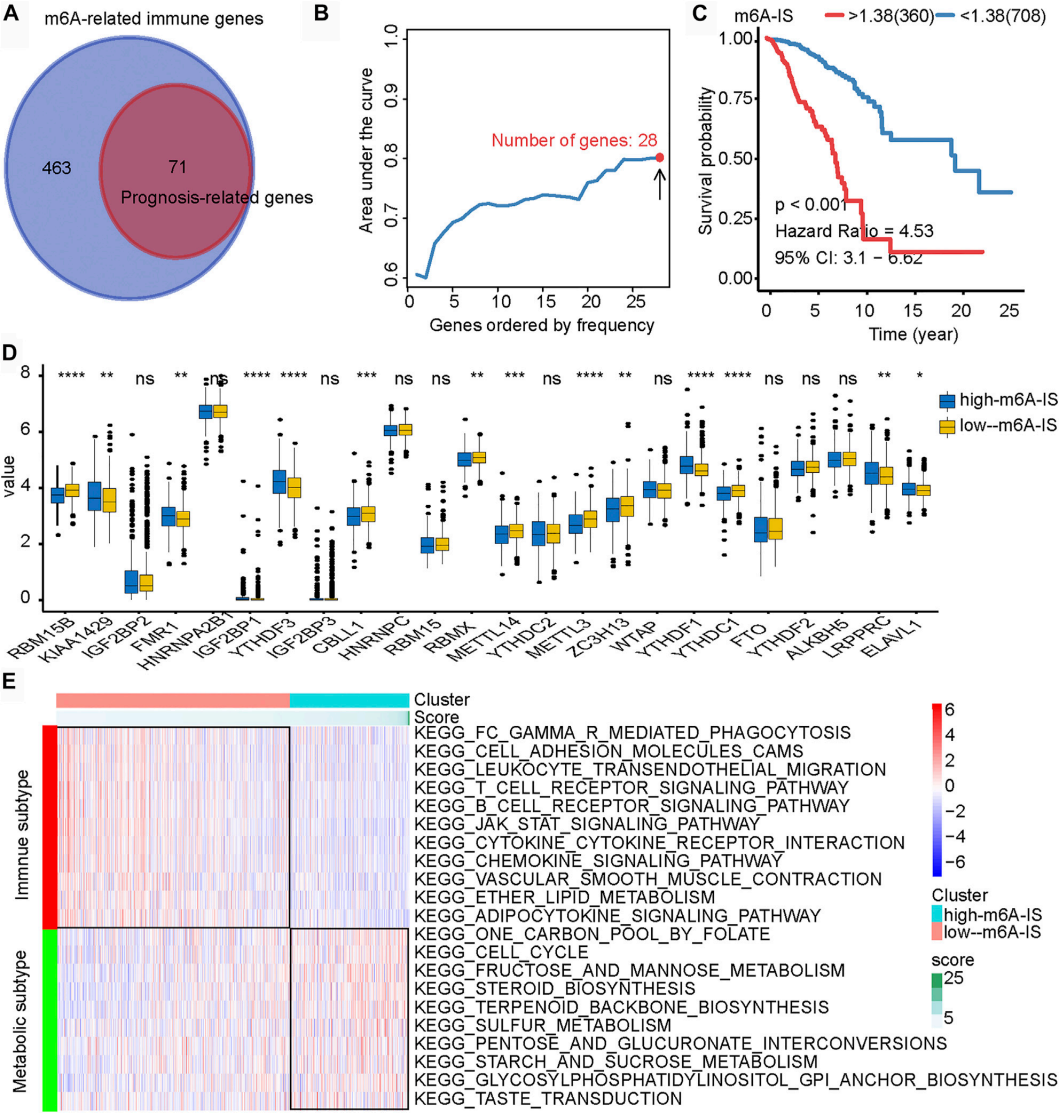
Construction of the *N* ^6^-methyladenosine-related immune score (m6A-IS) and analysis of its characteristics. **(A)** Through correlation analysis, a total of 534 tumor microenvironment (TME)-related genes were found to be significantly positively or negatively correlated with m6A regulators (|Pearson’s correlation coefficient| ≥ 0.5 and *p* < 0.001; Figure2A). Then, univariate Cox regression analysis further revealed that 71 TME-related immune genes were significantly related to the prognosis of breast cancer (*p* < 0.05; Figure2A). **(B)** The iterative LASSO algorithm was used to analyze a total of 71 TME-related genes. **(C)** The low m6A-IS subgroup showed longer survival than the high-m6A-IS subgroup (HR = 4.53, 95% CI = 3.04–6.66, *p* < 0.001). **(D)** Expressions of the 24 m6A regulators between the high- and low-m6A-IS groups of patients with breast cancer. High score, *blue*; low score, *yellow*. The *box bounds* represent an interquartile range of values, *center lines* represent the median value, and *black dots* show outliers. *Asterisks* represent the statistical *p*-value (*p < 0.05; **p < 0.01; ***p < 0.001). **(E)** Heatmap of the significantly enriched terms in the Kyoto Encyclopedia of Genes and Genomes (KEGG) pathways.

Correlation analysis was used for preliminary screening to further analyze which TME-related immune genes were related to m6A-regulated expression. Through correlation analysis, a total of 534 TME-related genes were found to be significantly positively or negatively correlated with m6A regulators (|Pearson’s correlation coefficient| ≥ 0.5 and *p* < 0.001) (Figure 3A). Univariate Cox regression analysis further revealed that 71 TME-related immune genes were significantly associated with breast cancer prognosis (*p* < 0.05; Figure 3A). Finally, 71 TME-related immune genes were included in the iterative LASSO algorithm for analysis. The results showed that, when these 28 TME-related immune genes were included (Supplementary Table S1), the model had the highest accuracy in predicting the prognosis (Figure 3B).

Multivariate Cox regression analysis was performed to construct the m6A-IS. The m6A-IS was expressed as follows: m6A-IS = (coefficient mRNA_1_ × mRNA_1_ expression) + (coefficient mRNA_2_ × expression of mRNA_2_) + …+ (coefficient mRNA_*n*_ × expression mRNA_*n*_). The results of the multivariate Cox regression analysis and m6A-IS are shown in Supplementary Tables S2 and S3. The results of the survival analysis showed that m6A-IS = 1.38 was the best cutoff value, and the low-m6A-IS group had a longer survival time in breast cancer than the high-m6A-IS group (Figure 3C). We also observed the same result from the METABRIC cohort (Supplementary Figure S1A).

To identify the signatures of the low- and high-m6A-IS subgroups, we analyzed the differences in the expressions of m6A modulators in the two subgroups and discovered that a large proportion of m6A regulators were differentially expressed in the high- and low-m6A-IS groups dramatically (Figure 3D). The clinicopathological information of the breast cancer patients has been shown in Table 1. The results of the Kyoto Encyclopedia of Genes and Genomes (KEGG) enrichment analysis showed that the low-m6A-IS group was mainly enriched in the activation of some immune pathways, indicating that patients in this group may be with immune subtypes (Figure 3E). The high-m6A-IS group was mainly enriched in glucose metabolism and lipid metabolism pathways, indicating that patients in this group may be with metabolic subtypes (Figure 3E).

**TABLE 1:** Clinical characteristics of the high- and low-m6A-IS groups

	**High m6A-IS (360)**	**Low m6A-IS (708)**	***p*-value**
Age (years)			
<65	236	510	0.0292
≥65	124	198	
T stage			
T1	60	219	<0.0001
T2	236	380	
T3	48	84	
T4	15	23	
Unknown	1	2	
N stage			
N0	173	329	0.1094
N1	107	249	
N2	44	76	
N3	32	41	
Unknown	4	13	
M stage			
M0	295	594	0.0363
M1	12	10	
Unknown	53	104	
Tumor stage			
Stage I	42	139	0.0010
Stage II	216	391	
Stage III	85	158	
Stage IV	12	8	
Unknown	5	12	
Subtype			
TNBC	16	94	<0.0001
Other	344	614	

### Relationship Between Clinicopathological Characteristics and *N* ^6^-Methyladenosine-Related Immune Score

**FIGURE 4:**
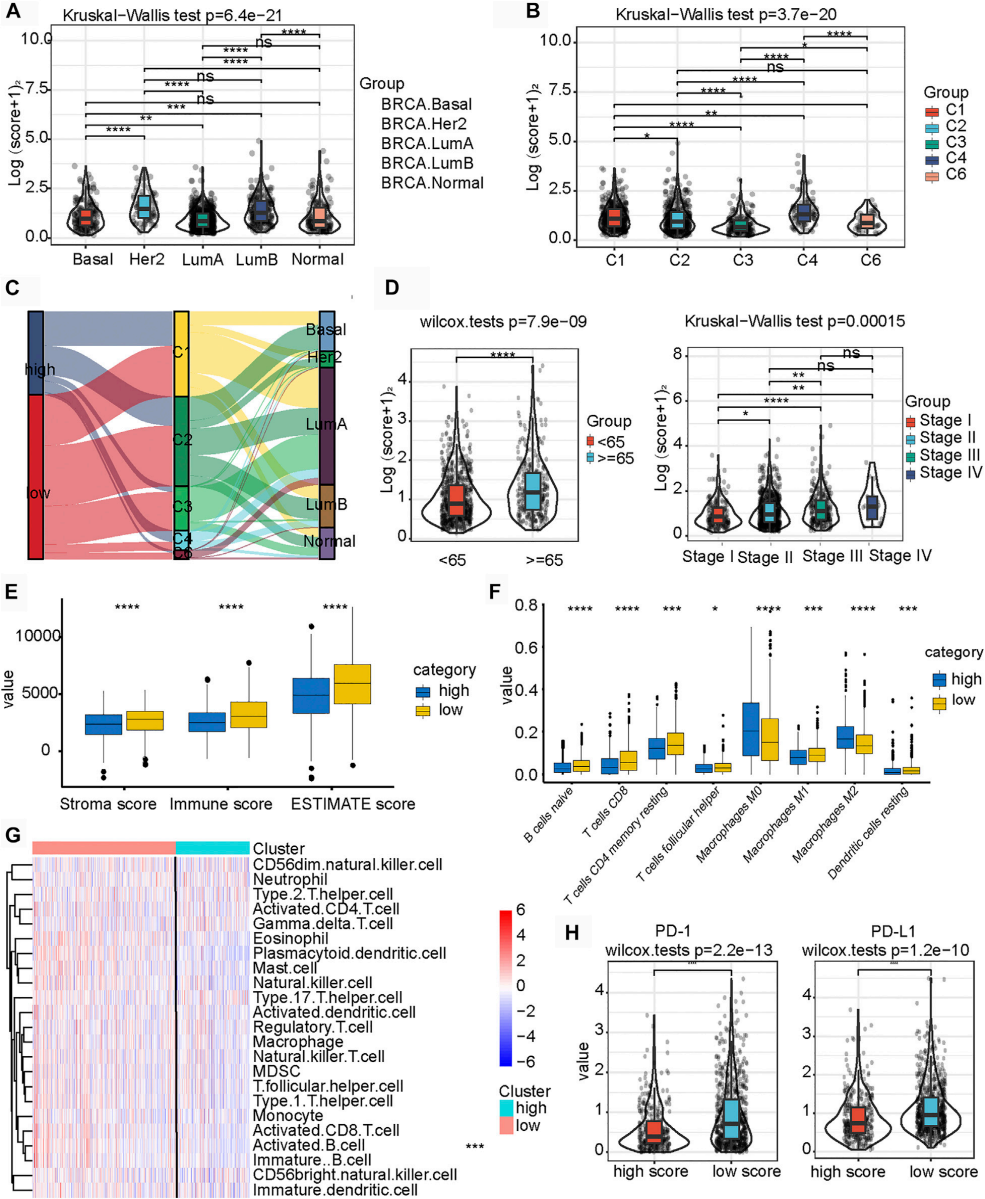
Relationship between clinicopathological characteristics and the *N* ^6^-methyladenosine-related immune score (m6A-IS). **(A)** Difference in m6A-IS in breast cancer with different molecular subtypes. Statistical difference in different breast cancer molecular subtypes was compared by the Kruskal–Wallis test (*p < 0.05; **p < 0.01; ***p < 0.001). **(B)** Difference in m6A-IS between different immunogenomic subtypes. The Kruskal–Wallis test was performed to compare the statistical difference in each immunogenomic subtype (*p < 0.05; **p < 0.01; ***p < 0.001). **(C)** Alluvial diagram representing the association of m6A-IS, six immune subtypes, and clinical molecular subtypes in breast cancer. **(D)** Breast cancer patients with different clinicopathological features had different expression levels of m6A-IS (*p < 0.05; **p < 0.01; ***p < 0.001). **(E)** Difference between the stroma score, the immune score, and the ESTIMATE score in the high- and low-m6A-IS groups (*blue* represents the high-m6A-IS group and *yellow* the low-m6A-IS group) (*p < 0.05; **p < 0.01; ***p < 0.001). **(F)** Difference in the abundance of 8 types of infiltrating immune cells between the high- and low-m6A-IS groups (*blue* represents the high-m6A-IS group and *yellow* the low-m6A-IS group) (*p < 0.05; **p < 0.01; ***p < 0.001). **(G)** Heatmap of the tumor-infiltrating cell proportions between the high- and low-m6A-IS groups. **(H)** Expression levels of PD-1/PD-L1 between the low- and high-m6A-IS groups (Wilcox test: *p* < 0.0001).

We investigated the expression levels of m6A-IS from the five breast cancer subtypes previously reported to further analyze its characteristics (Parker et al., 2009). We found that the Her-2 and luminal B subtypes had the highest m6A-IS, followed by the basal and normal subtypes. The luminal A subtype had the lowest m6A-IS level (Figure 4A and Supplementary Figure S1B). This is consistent with the results of previous studies: luminal A subtype has the best prognosis, Her-2 and luminal B subtypes have the worst prognosis, and the prognosis of the basal and normal subtypes can be characterized at a level between the best and worst. We classified the breast cancer samples based on the six immune subtypes previously identified using immunogenomic features (Thorsson et al., 2018). The results showed that m6A-IS expression was the lowest in the C3 subtype; meanwhile, it was the highest in the C4 subtype. This result suggests that the C3 subtype had the best prognosis, while the C4 subtype had the worst prognosis (Figure 4B). Thorsson et al. discovered that the C4 subtype displayed high M2 macrophage domination and low lymphocytic infiltrate, which induced poor outcomes. In contrast, the C3 subtype, which showed a type I immune response and remarkable Th17 signature, had favorable prognosis. Our results are in line with the conclusions of a previous report (Thorsson et al., 2018), indicating that the m6A-IS has good robustness in different breast cancer molecular subtypes. A Sankey diagram was used to visualize the relationship between m6A-IS and the two breast cancer subtypes (Figure 4C). In addition, between-group comparisons of age were performed using the Wilcoxon test, and there was a significant difference in the distributions of age. Patients older than 65 years were more likely to have higher scores than patients aged 65 years or younger (Figure 4D and Supplementary Figure S1C). Interestingly, we found that, with increased malignancy, the m6A-IS showed a significant stepwise increase in non-metastatic breast cancer; meanwhile, no significant increase was observed in patients with metastatic breast cancer (stage IV) (Figure 4D).

These results indicate that m6A-IS can be used to characterize the existing subtypes and clinical features of patients with breast cancer. However, the relationship between m6A-IS and TME remains unknown. For this reason, we found through ssGSEA that the low-m6A-IS group had a higher expression of stromal and immune scores than the high-m6A-IS group (Figure 4E), which is consistent with previous results (Figure 3E). In addition, the CIBERSORT algorithm (Figure 4F) and the enrichment analysis of 23 immune cells (Figure 4G and Supplementary Figure S1D) showed that the infiltration levels of B cells, CD8 T cells, and M1 tumor-associated macrophages in the low-m6A-IS group were significantly higher than those in the high-m6A-IS group. In contrast, the infiltration level of M2 tumor-associated macrophages increased significantly in the high-m6A-IS group. Finally, we found that the expression levels of the immune checkpoint molecules (PD1 and PD-L1) in the low-m6A-IS group were significantly higher than those in the high-m6A-IS group (Figure 4H). These results suggest that the low-m6A-IS group had increased immune cell infiltration and, simultaneously, had increased immunosuppression.

### Potential Immune Escape Mechanisms in High and Low *N* ^6^-Methyladenosine-Related Immune Score

**FIGURE 5:**
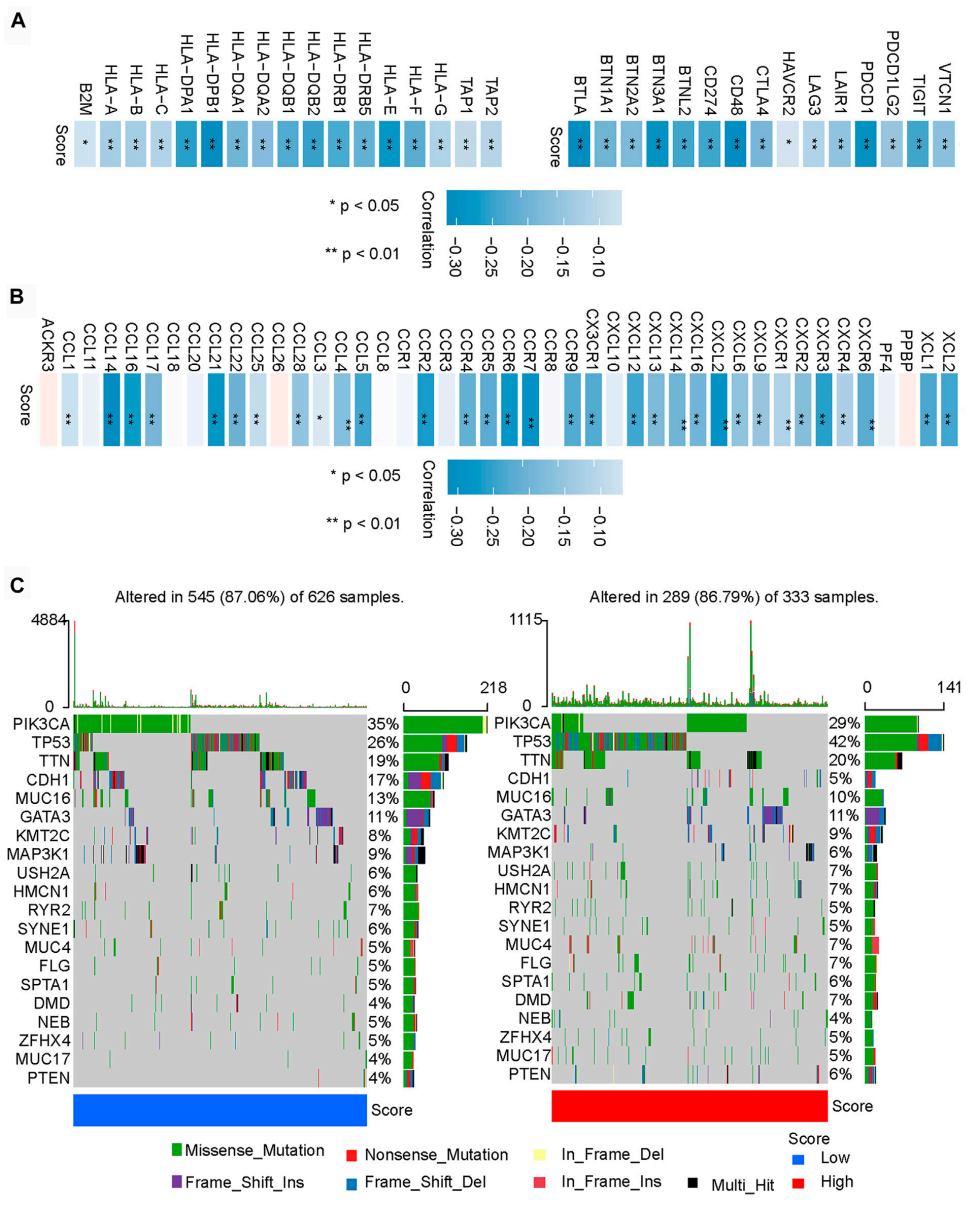
Potential immune escape mechanisms in high and low *N* ^6^-methyladenosine-related immune scores (m6A-IS). **(A)** Correlation analysis between the expressions of MHC molecules and co-inhibitory molecules and m6A-IS (*p < 0.05; **p < 0.01; ***p < 0.001). **(B)** Correlation analysis of the expressions of chemokines and m6A-IS (*p < 0.05; **p < 0.01; ***p < 0.001). **(C)** Waterfall plot of tumor somatic mutations in the low- ***(left)*** and high-m6A-IS ***(right)*** groups. Individual patients are represented in each column. Missense mutation, *green*; nonstop mutation, *grey*; nonsense mutation, *red*; multi-hit, *black*. The *right bar plot* shows the mutation frequency of each gene in separate subgroups.

We further compared the two groups and found that the low-m6A-IS group had a higher expression of major histocompatibility complex (MHC)-related antigen-presenting molecules than the high-m6A-IS group (all *p* < 0.05; Figure 5A). We analyzed some co-inhibitory molecules to compare their expressions in the high and low-m6A-IS groups, and the results showed that the expression levels of these molecules were significantly correlated with m6A-IS. The results showed that the low-m6A-IS group had higher expressions of immunosuppressive molecules than the high-m6A-IS group (all *p* < 0.05; Figure 5A). Further analysis revealed that the low-m6A-IS group showed higher chemokine expressions (Figure 5B). These results indicate that the low-m6A-IS group had higher immunogenicity than the high-m6A-IS group. However, at the same time, there was a significant immunosuppressive state in the low-m6A-IS group. This suggests that there is a potential immune escape mechanism in low-m6A-IS.

Finally, the top 20 genes were further analyzed with the highest mutation frequency in the high and low-m6A-IS groups (Figure 5C). The results showed that *PIK3CA* and *CDH1* had increased mutation frequencies in the low-m6A-IS group, while *TP53* had an increased mutation frequency in the high-m6A-IS group. These results suggest that *PIK3CA* and *CDH1* mutations may be related to a high immune infiltration, while *TP53* mutations may be related to immunosuppression, which may require further analysis.

### *N* ^6^-Methyladenosine-Related Immune Score Was an Independent Prognostic Factor for Patients With Breast Cancer

**FIGURE 6:**
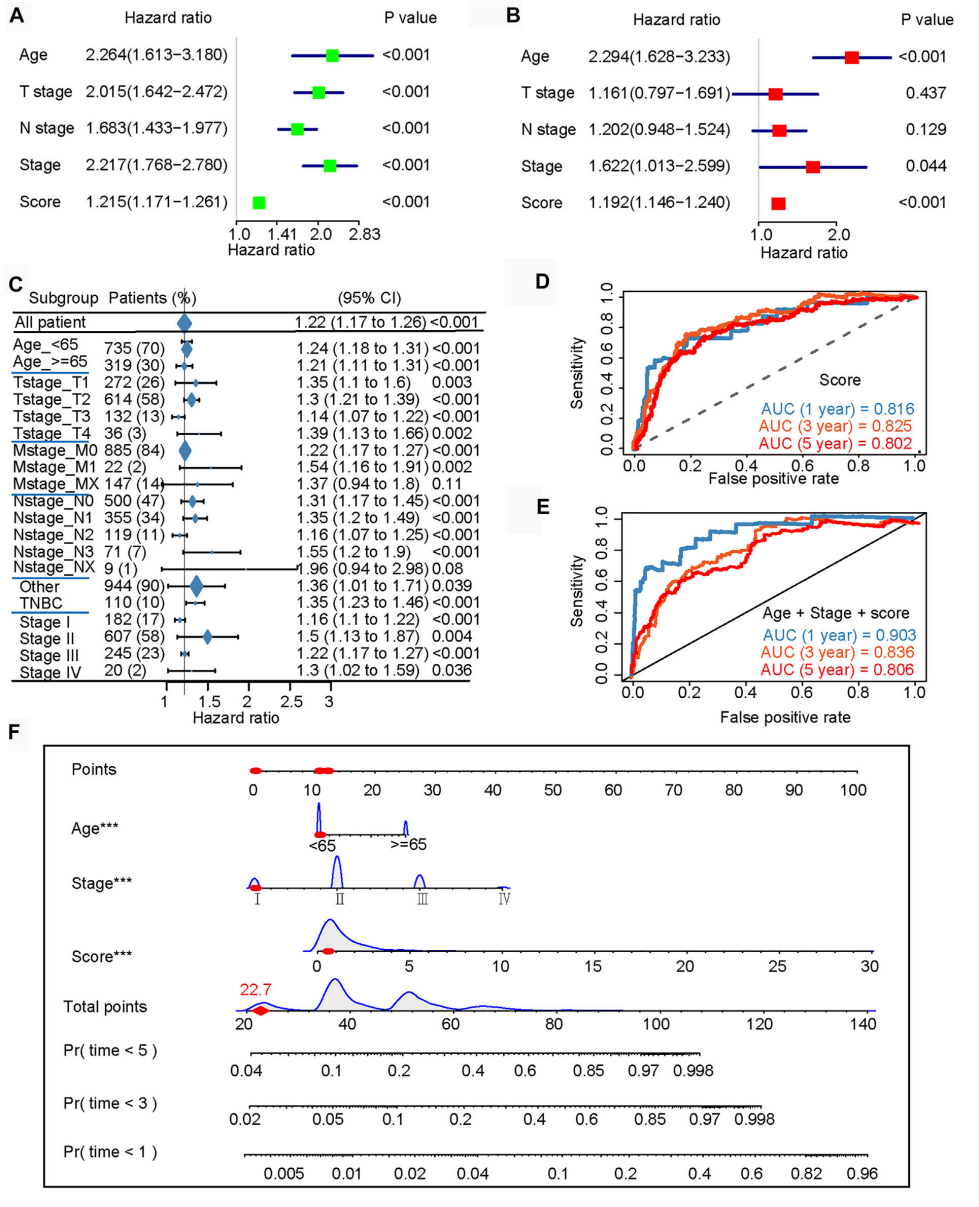
*N* ^6^-methyladenosine-related immune score (m6A-IS) is an independent prognostic factor for patients with breast cancer (BRCA). **(A**,**B)** Univariate and multivariate Cox analyses further showed that age, tumor stage, and m6A-IS are independent prognostic predictors for patients with BRCA. **(C)** Univariate Cox regression analysis of the overall prognostic value of m6A-IS in each clinical feature subgroup. **(D)** ROC curve constructed based on m6A-IS. The AUCs of the ROC curve at 1, 3, and 5 years were 0.816, 0.825, and 0.802, respectively. **(E)** The m6A-IS combines age and tumor stage to construct a final predictive prognostic model. The results showed that the multi-predictor ROC has excellent accuracy for 1-, 3-, and 5-year overall survival (OS) (AUC = 0.903, 0.836, and 0.806, respectively). **(F)** Nomogram based on m6A-IS, age, and stage in The Cancer Genome Atlas (TCGA) cohort.

Univariate and multivariate Cox analyses represented that age, tumor stage, and the m6A-IS were independent prognostic predictors for patients with breast cancer (Figures 6A, B). Subsequently, we conducted a more detailed stratification of breast cancer patients based on clinical characteristics. Univariate Cox regression analysis revealed that m6A-IS has a good prognostic value in each subgroup (Figure 6C). Furthermore, the triple-negative (TNBC) subtype has the strongest tumor immunogenicity of all BC subtypes (Liu et al., 2018); meanwhile, m6A-IS can also be used to assess prognosis in this subtype (Figure 6C). These results indicate that m6A-IS, as an independent prognostic indicator, may be useful for clinical prognosis evaluation.

An ROC curve was constructed based on the m6A-IS. We found that the AUCs of the ROC curve at 1, 3, and 5 years were 0.816, 0.825, and 0.802, respectively (Figure 6D). Next, we verified whether the m6A-IS can predict the prognosis of breast cancer patients in the METABRIC cohort (Supplementary Figure S1E). It was shown that m6A-IS has good ability to predict prognosis. The previous results found that age and tumor stage were also independent factors; therefore, we combined them with the m6A-IS to build the final predictive prognosis model (Figure 6E). The results showed that the multi-predictor ROC had excellent accuracy for 1-, 3-, and 5-year OS (AUC = 0.903, 0.836, and 0.806, respectively). Based on the results of the logistic regression analysis and the ROC curves, a nomogram was graphically depicted (Figure 6F). By calculating the total scores of each selected variable, the survival of individual breast cancer patients at 1, 3, and 5 years can be easily estimated by plotting a vertical line between the total points and each prognosis axis of the nomogram.

### Role of the *N* ^6^-Methyladenosine-Related Immune Score in Anti-PD-L1 Immunotherapy

**FIGURE 7:**
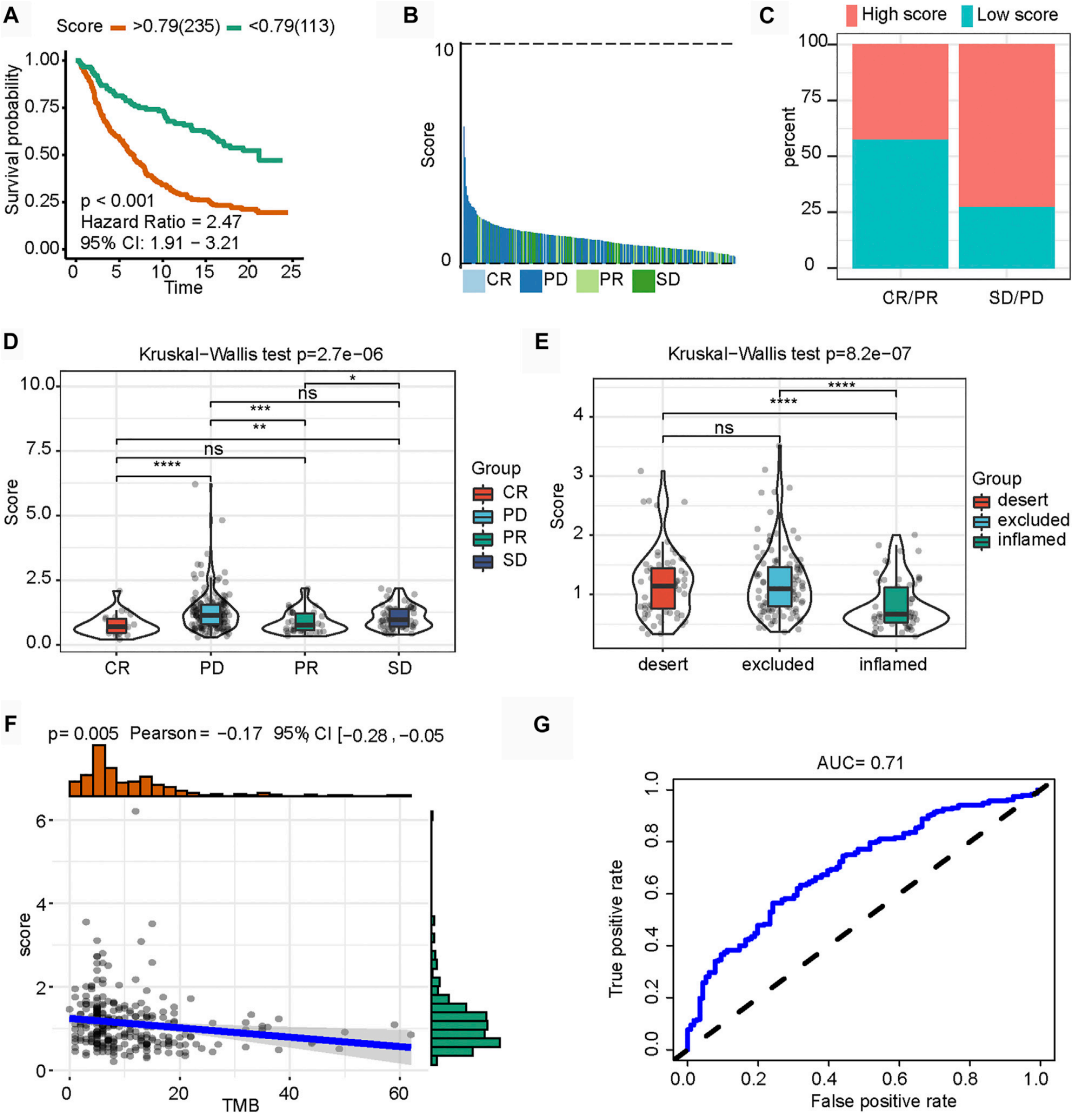
Role of *N* ^6^-methyladenosine-related immune score (m6A-IS) in immunotherapy. **(A)** Patient survival analysis defined by the high- (*n* = 235) and low-m6A-IS (*n* = 113) groups based on the IMvigor210 cohort (log-rank test: *p* < 0.001). **(B)** Waterfall plot of m6A-IS for distinct clinical response groups based on the IMvigor210 cohort. **(C)** Stacked bar chart representing the percentage of clinical response patients assigned to two m6A-IS subgroups in the IMvigor210 cohort. High-score, *red*; low score, *blue*. **(D)** Boxplot of m6A-IS for distinct clinical response groups *via* the Kruskal–Wallis test (*p* < 0.001). **(E)** Differences in m6A-IS among three distinct tumor immune phenotypes in the IMvigor210 cohort by the Kruskal–Wallis test (*p* < 0.001). **(F)** Spearman’s correlation analysis between m6A-IS and tumor mutation burden (*p* = 0.005). **(G)** Predictive value of the quantification of m6A-IS in patients treated with anti-PD-L1 immunotherapy (AUC = 0.71).

**FIGURE 8:**
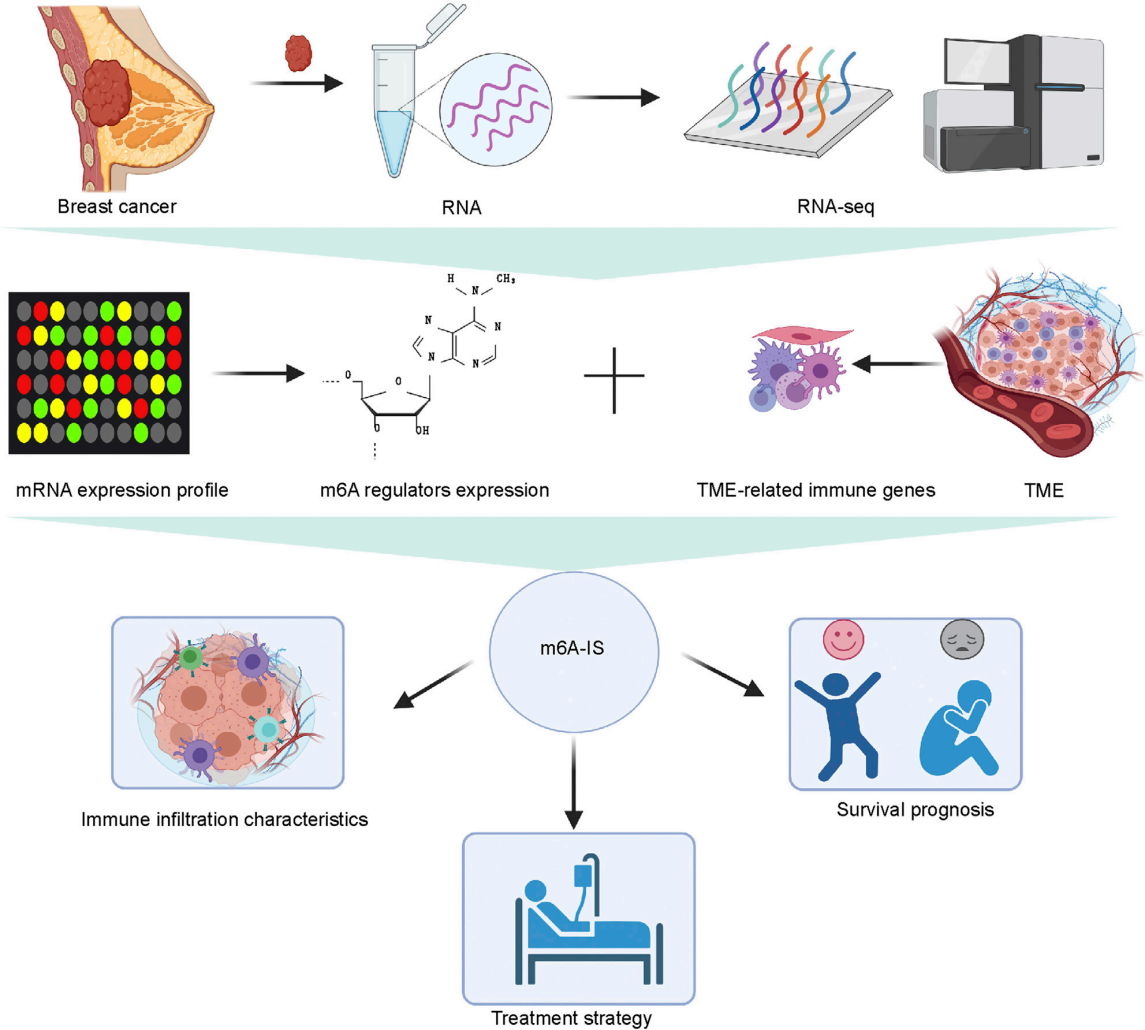
Illustration for this study.

Recently, the connection of m6A modification with PD-L1 expression has been reported. In an anti-PD-L1 cohort (IMvigor210 cohort), the low-m6A-IS group exhibited significant clinical benefits and a markedly longer survival time (Figure 7A). Compared with those with high m6A-IS, patients with low m6A-IS showed significant survival advantages and clinical response to anti-PD-L1 therapy (Figures 7B–D). Next, we examined the differences in m6A-IS among the different immune phenotypes in the IMvigor210 cohort. Interestingly, patients in the higher m6A-IS group were remarkably relevant to the exclusion and desert immune phenotypes, and it was difficult to achieve antitumor effects of the checkpoint inhibitors in these phenotypes (Figure 7E). Based on accumulating evidence, high levels of tumor mutation burden (TMB) and PD-L1 expression have long-lasting clinical responses to immunotherapy. A high TMB is generally considered to be a preexisting adaptive immune response to the tumor, and patients with a high TMB who received PD-1 blocking immunotherapy showed an improved response and enhanced clinical efficacy compared to patients with a moderate or low TMB (Hellmann et al., 2018). Further analysis revealed that m6A-IS was significantly negatively correlated with TMB (Figure 7F). These results suggest that the quantification of the modification patterns of m6A is a potential and robust biomarker for the assessment of prognosis and clinical response to immunotherapy (Figure 7G). In conclusion, this study emphasizes that m6A methylation modification is significantly correlated with tumor immunophenotype and anti-PD-L1 immunotherapy response, and established m6A modification characteristics may help predict the response to anti-PD-L1 immunotherapy. An illustration of this study is shown in Figure 8.

## Discussion

Breast cancer is characterized by high morbidity and ranks first among female malignant tumors globally (Harbeck et al., 2019). Within the genomic heterogeneity and diverse histological features, patients with breast cancer present individual responses to traditional treatments, including surgery, chemotherapy, hormonal therapy, and target therapy (Colozza et al., 2007; Foukakis et al., 2011). Immunotherapy combined with chemotherapy is emerging as a novel treatment regimen for breast cancer (Adams et al., 2019b). Although breast cancer is considered less immunogenic, lower mutational load than other solid carcinomas, the synergism between anti-PD1/PD-L1 agents and chemotherapy has been supported *via* multiple preclinical pieces of evidence, particularly in the TNBC subtype (Adams et al., 2019a; Adams et al., 2019c; Loibl et al., 2019; Planes-Laine et al., 2019). Similarly, more studies are needed to identify novel immunotherapy result prediction.

Sample classification methods based on a predefined multi-gene signature is a proven approach for predicting the treatment benefits of immune checkpoint inhibition in a variety of malignancies (Cristescu et al., 2015). Dysregulation of m6A methylation has been shown to be closely associated with the antitumor immune dysregulation through interactions with various m6A regulators (Wu et al., 2019; Yang et al., 2019; Wang et al., 2020b). In this study, we screened and identified 24 m6A regulators and drew a gene signature based on the dataset from TCGA. A prognostic model (m6A-IS) was constructed to correlate with the clinical outcomes of breast cancer patients by combining the roles of immune infiltrating cells in the TME with these m6a-related genes.

In our study, we constructed the m6A-IS to quantify the prognostic outcomes based on two groups (high and low), providing strong evidence for individualized immunotherapy in breast cancer. Indeed, the m6A-IS reflects the heterogeneity of patients. Secondly, the model links m6A methylation to the prognosis of breast cancer immunotherapy. The m6A-IS score includes, but is not limited to, regulatory factors related to m6A, such as *HNRNPC*, *RBM15B*, and *YTHDF3*. *HNRNPC* appeared to be related to good outcomes in glioma patients (Wang et al., 2020a) and was associated with an increased proportion of patients at low risk of lung squamous cell carcinoma based on an immune-related prediction model (Xu et al., 2020) that is similar to our model. However, Wu et al. found that aberrant upregulation of *HNRNPC* resulted in the accumulation of endogenous double-stranded RNA and tumorigenesis in breast cancer cell lines (Wu et al., 2018). Indeed, more samples are needed to draw more precise conclusions. The methyltransferase *RBM15B* is a paralog of *RBM15* and can be involved in regulating immunological phenotypes. High *RBM15B* levels are correlated with multiple immune signatures and cancer-related pathways (Fang et al., 2020). It has been reported that the m6A reader *YTHDF3* promotes ribosome loading with *YTHDF1*, and a high *YTHDF3* expression in breast cancer clinically correlates with brain metastases (Chang et al., 2020). However, further studies are needed to determine whether these genes can be new targets for improving the response to immunotherapy. Additionally, the m6A-IS represents patients with different clinical characteristics and is related to immunotherapy. High grouping showed an m6A modification pattern characterized by an immune-excluded phenotype, suggesting worse clinical features and a lower predicted survival time. The pattern characterized by the immune-inflamed phenotype showed lower m6A-IS. Moreover, the infiltration of TME cells indicates that m6A-IS is important for immunotherapy. In low-group patients, the upregulation of immune cell infiltration associated with immune activation correlated with improved prognosis for immunotherapy. For example, a study showed that naive B cells and CD8^+^ T cells appeared to be anticancer immune cells (Zhang et al., 2019; Bu et al., 2020); meanwhile, M2 macrophages, immune cells that promote tumor proliferation and metastasis, were increased in this study (Tariq et al., 2017). Interestingly, clinical trials have shown promising prospects for immunotherapy in patients with the TNBC subtype. In our predicted model, the TNBC subtype in patients was significantly associated with lower m6A-IS. Moreover, TMB is a determinant of the immune-mediated survival of breast cancer patients and can be an independent predictor of immunotherapeutic response in various cancers (Goodman et al., 2017; Thomas et al., 2018; Lee et al., 2019). Our data revealed a significant negative correlation between the m6A score and TMB. Additionally, m6A-IS may also be used for other tumors and immune-related tumors. Researchers have studied prognostic models of breast cancer immunotherapy under modified conditions in hypoxia, ferroptosis, or autophagy (Lin et al., 2020; Zheng et al., 2020; Tang et al., 2021). In our research, we focused on exploring the extensive regulatory mechanism of m6A methylation modification in the breast cancer microenvironment. Thus, our model is valuable in facilitating breast cancer treatment. In addition, these results were validated in the IMvigor210 cohort with a defined immunophenotype. This indicates that m6A-IS has a predictive advantage for selecting the appropriate immunotherapy for breast cancer. Moreover, our model can be used to further determine the TME infiltration pattern, which is a tumor immunophenotype.

Despite conducting multi-pronged and multi-database verification, our study has several limitations. Firstly, our prognostic model needs to be further validated using forward-looking, multicenter, real-world data. In addition, the underlying mechanism between m6A regulators and TME needs to be further tested *via* clinical molecular experiments of the potential molecular mechanism of the breast cancer immunotherapy response. The results of single-cell sequencing should contribute to an increased understanding of the specific changes in the TME, which is also an aspect of our future concern. These not only increase the challenges but also motivate us to conduct future research.

## Conclusion

We constructed the m6A-IS to assess the prognosis of patients suffering from breast cancer. Patients with low m6A-IS had a longer survival time. The results of our study provide insights into the mechanism of immune infiltration and immune evasion in breast cancer based on m6A-IS stratification. The m6A-IS was used to stratify patients and determine those who will gain a survival benefit from immunotherapy, thereby contributing to improved diagnosis and treatment of breast cancer.

## Data Availability

The original contributions presented in the study are included in the article/Supplementary Material. Further inquiries can be directed to the corresponding authors.
