# Supplementary figures and images for: N6-methyladenosine Regulator-Mediated Immune Genes Identify Breast Cancer Immune Subtypes and Predict Immunotherapy Efficacy
Source: Front Genet. 2021 Dec 17;12:790888. doi: 10.3389/fgene.2021.790888 (PMC8718791; doi:10.3389/fgene.2021.790888)

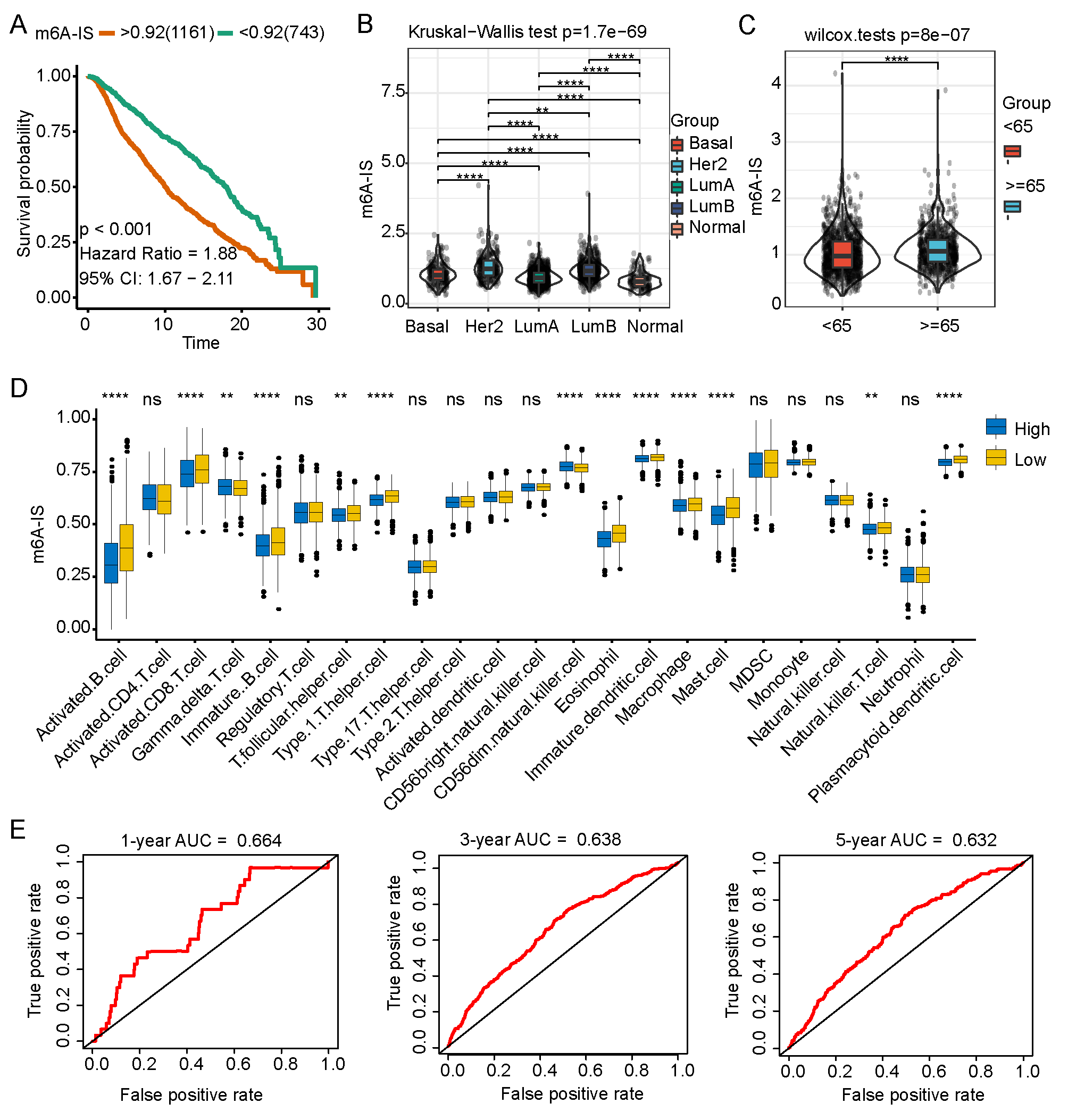

Supplement: Supplementary file 4 [file Image1.TIF]
